# To BSE or not to BSE: a capital budgeting analysis of the use of the bull breeding soundness evaluation (BSE) to improve reproductive efficiency and profitability in cow-calf herds

**DOI:** 10.1093/tas/txaf024

**Published:** 2025-03-04

**Authors:** Todd G Gunderson, Kevin N Kim, Kalyn T Coatney, David R Smith

**Affiliations:** Department of Clinical Sciences, Kansas State University, Manhattan, KS 66506, USA; Department of Agricultural Economics, Mississippi State University, Mississippi State, MS 39762, USA; Department of Agricultural Economics, Mississippi State University, Mississippi State, MS 39762, USA; Department of Pathobiology and Population Medicine, Mississippi State University, Mississippi State, MS 39762, USA

**Keywords:** breeding, budget, bull, capital, financial, soundness

## Abstract

The breeding soundness evaluation (BSE) as defined by the Society for Theriogenology is intended to identify subfertile bulls. Removing subfertile bulls and replacing them with fertile bulls is expected to produce financial benefits in the form of more pregnant cows within a designated breeding season and subsequently higher weaning rates. However, past survey results of cow-calf producers indicate low adoption rates of the BSE. To better understand the rationale of these decisions by producers, a financial evaluation by means of a capital budgeting model was developed to calculate the net present value (NPV) of two different herd management strategies; one that performs BSE to one that does not. Because there are various sources of uncertainty in cattle production, a Monte Carlo simulation analysis was employed to estimate the differences between the expected NPV of these two strategies. Simulations were conducted across a range of plausible fertility differences from using BSE and revenues were generated from the resulting calf outputs and herd replacements. Additionally, the length of the breeding season and the cow:bull ratio were varied to capture a typical range of heterogeneous production systems. For each scenario considered, the results indicated that the likelihood of improving profitability by performing BSE increases as breeding season length decreases and cow:bull ratios increase, despite the relative increase in associated costs from hiring veterinarians to perform BSE. These results are largely driven by the increase in total weight of calves weaned and a decrease in costs associated with the replacement of non-pregnant cows. Overall, these findings provide a plausible financial explanation for why cow-calf producers with different management strategies are more/less willing to perform BSE.

## Teaser

Despite its long history and widespread promotion by animal health professionals, only a minority of cow-calf producers use breeding soundness evaluations to screen their herds for subfertile bulls. This study puts forth financial reasoning for this observation, specifically identifying the influence of breeding season length and cow to bull ratio on the profitability of performing breeding soundness evaluations.

## Graphical Abstract

**Figure:**
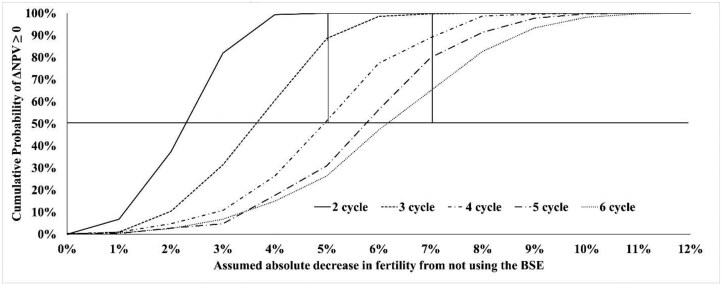
Fig. 2. The cumulative probability curves for the likelihood that a strategy that uses breeding soundness evaluations (BSE) to remove subfertile bulls from the herd will have a higher net present value (NPV) than a strategy that does not. Each curve represents a different breeding season length in units of estrus cycles and assumes that both strategies use a cow:bull ratio of 25:1. The vertical lines represent differences in fertility of 5% & 7%, which is approximately the range of expected differences in incidence rate of conception per cycle between herds that use BSE and those that don’t based on published literature.(Wiltbank and Parish, 1986 DOI: 10.1016/0093-691X(86)90093-2) The horizontal line represents the point at which it is equally likely that using the BSE will have a higher NPV than not using the BSE.

## INTRODUCTION

The breeding soundness evaluation (BSE) has been used by veterinarians and animal scientists for several decades to screen bull batteries for subfertile bulls.(Bartlett, n.d.) In the U.S., standards for performing BSE have been published by the Society for Theriogenology (SFT).(Chenoweth et al., 1994; Koziol and Armstrong, 2018) It has been suggested that these standards are intended to assess the likelihood of an individual bull impregnating 25 or more healthy, cycling females in a 65 to 70 d breeding season.(Kastelic and Thundathil, 2008) To the best of the authors’ knowledge, this is not an official position of SFT. However, this is a useful standard for conceptualizing the difference between a fertile and subfertile bull. A subfertile bull may be able to impregnate females, but he is not considered fertile unless he can impregnate a certain number of females within a certain amount of time corresponding to what could reasonably be expected for a male of his species and breed.

Recent trends indicate that U.S. cattle producers have encountered increasing challenges in generating profits. According to a survey conducted by the USDA Economic Research Service, the average operating profit margin for beef cattle producers between 2019 and 2023 was −22.1%.(United States Department of Agriculture, 2023) This prolonged period of negative profitability differentiates beef cattle producers from other livestock producers, such as those in poultry, hog, and dairy production, as well as commodity producers, which consistently maintained positive profit margins during the same time period.(United States Department of Agriculture, 2023) Additional measures of profitability, including return on assets and return on equity, similarly indicate negative outcomes for cattle producers over the years. Sustained negative profitability in a business can adversely affect land value, increase the cost of raising additional capital, and threaten the survival of the underlying business. Therefore, it is crucial to pursue changes in practices that can reduce profitability uncertainties and/or enhance efficiency.

Improving reproductive efficiency is one of the most impactful ways to improve profitability.(Pendell and Herbell, 2021) Multiple studies have demonstrated benefits to reproductive efficiency by screening bulls using the BSE.(Makarechian and Farid, 1985; Wiltbank and Parish, 1986; Coulter and Kozub, 1989; Farin et al., 1989; Fitzpatrick et al., 2002; Whitworth et al., 2008) Menegassi et al. (2011) evaluated these benefits relative to the cost of implementing BSE in groups of Charolais and Charolais cross cattle in Brazil over a 3 yr period and found that investing in the practice of performing BSE yielded a benefit:cost ratio of ~36:1. The herds that implemented BSE in this region utilized higher cow:bull ratios than the herds that did not, and also experienced dramatic increases in prolificacy (i.e., the ratio of calves to cows), especially after the first year. The study accounted for an increase in bull culling rates in herds that implemented the BSE, though the cull rate was higher in the first year than in subsequent years. The study did not specify the breeding season lengths for any of the herds involved, but it was implied from their results that the herds that implemented BSE had more cows impregnated earlier in the breeding season based on the increase in average weaning weight. The study also cited several other studies that demonstrated favorable benefit:cost ratios for using the BSE.(Menegassi et al., 2011)

Despite the identified benefits of performing the BSE, Menegassi et al. (2011) reported that only about 10% of breeders in the region surveyed used BSE.(Menegassi et al., 2011) Data in the U.S. for 2017 indicate that only about 20% of producers use BSE (i.e., semen test their bulls).(National Animal Health Monitoring System, 2020) Recent survey results for the state of Mississippi found that cow-calf producers who did not have a defined breeding season (i.e., they leave their bull(s) out with their cows year around) had lower odds of performing BSE.(Gunderson et al., 2024) These observations have prompted the question, “given the apparent benefits of the BSE, why do some cow-calf producers choose not to use it to manage reproductive efficiency in their herds?” If the goal of performing BSE is to identify bulls that are not capable of impregnating a specific quota of fertile females within a defined breeding season, it is reasonable to conclude that producers who do not have a defined breeding season may not perceive a financial benefit from performing BSE. Alternatively, rather than use BSE to screen for subfertile bulls, a producer who wishes to maximize reproductive efficiency may choose to run more bulls in a pasture to decrease the fertility burden on each bull and dilute the effect a subfertile bull may have on overall fertility.

The purpose and major contribution of this study is the identification of plausible financial reasons why producers with prolonged, or poorly defined breeding seasons or varying cow:bull ratios may or may not choose to perform BSE. To do so, we develop financial capital budgeting models to compare the discounted net present values (NPV) between performing BSE or not, while accounting for various stochastic events throughout the planning horizon. Additionally, a series of sensitivity analyses are conducted under various production settings by varying breeding season length and cow:bull ratio, and evaluating the effect of these variations on the expected NPV over a range of plausible differences in fertility due to using, or not using the BSE.

## MATERIALS AND METHODS

No animals were used in this research. As such, it did not require approval by an institutional animal care and use committee. An overview of the methodology and assumptions to generate the financial comparisons of BSE management strategies is as follows. The basic production method evaluated is a cow-calf operation in the southeastern United States that turns bulls out to breed on the same date every year. All calves produced in the herd are weaned and sold 225 d after the scheduled start date for the calving season. For simplicity, the operation does not keep or develop any replacement heifers or other weaned calves; all replacements are purchased annually as heifers bred to calve within the first two estrus cycles (42 d) of the calving season. All cows in the herd are examined for pregnancy after every breeding season, and any cows that are diagnosed as non-pregnant are sold as culls. The operation has the capacity to maintain 1,000 mature (have had at least 1 calf), female cattle, and for each period of the model, the cow herd (CH) is held constant at 1,000 cows through the purchase of bred females. Though 1,000 is much larger than the typical cow herd in the Southeast, the magnitude selected acts as a scalar allowing for whole number bull replacement estimates. Also, the magnitude could be thought of as a consortium of smaller similarly situated producers. The number of bulls in the bull herd (BH) is determined by the cow:bull ratio (C:B) such that the number of bulls is $BH=\frac{CH}{C:B}$. The purchase price for the bulls is based on sale data of virgin Brangus or predominately Brangus bulls in the Southeast.

The financial model evaluates two different strategies for managing the bull battery. The first strategy involves hiring a veterinarian to perform BSE on bulls (BSE strategy), and the second strategy involves not hiring a veterinarian to perform this test (NBSE strategy). The financial analysis for each strategy consists of a 50-yr producer planning horizon from which long-term strategies can be compared. Each year represents one time-period in the financial analysis. We use 50 time periods (T) because it provides the maximum feasible evaluation of the long-term benefits to each strategy and is a close approximation to the maximum working life span of a cow-calf producer.

Estimates for revenues are based on number of cows conceiving in each estrus cycle (i.e., incidence rate of conception per cycle), the number of calves born and weaned from each of these cycles, average weaning weights of these calves, and number of cows and bulls culled. The number of cows calving and the weaning weights of calves each year are a function of bull fertility, breeding season length, and eligibility of cows to breed in any given estrous cycle. Estimates for costs are based on the maintenance costs for bulls and the number of cows and bulls needed to be purchased each year as replacements for culls and dead animals. Maintenance costs for cows are assumed to be equivalent between strategies and are not factored into the model. The budget parameters used in the financial model are presented in Table 1.

**Table 1.** Budget parameters for bulls, cows, and calves used in the capital budgeting model

Bull Parameters		Cow Parameters		Calf Parameters	
Cost to maintain 1 bull per year, $^1^	542.30	Cost to purchase 1 bred heifer, $^5^	1116	Average birth weight, Lb (kg)	80 (36)
Fee for BSE, $^2^	100	Cull cow weight, Lb (kg)	1000 (455)	Average daily gain, Lb/day (kg/day)	2 (0.9)
Average purchase price, $^3^	7,250	Cull cow price, $/Lb^6^ ($/kg)	0.86 (1.89)		
Cull bull weight, Lb (kg)	2000 (909)	Average useful life span, years	10		
Cull bull price, $/Lb^4^ ($/kg)	0.85 (1.87)				
Average useful life span, years	4				

^1^Courtesy of Bull Cost Calculator provided by Dr. Josh Maples, Mississippi State University Department of Agricultural Economics.

^2^Set to the expected high end of a what a producer might pay for a BSE.

^3^Based on sale data from 2023-2024, source: International Brangus Breeders Association.

^4^Aggregate weekly average MS slaughter bulls 2023, source: USDA/Agricultural Marketing Service (AMS).

^5^Aggregate weekly average MS bred heifers 2023, source: USDA/AMS.

^6^Aggregate weekly average MS slaughter cows 2023, source: USDA/AMS.

To compare strategies, we use a capital budgeting model that generates the NPV over the planning horizon, accounting for various sources of revenues and costs of production unique to each management strategy (e.g., veterinarian costs related to the BSE are not included in the NBSE strategy). Net present value discounts each future cash inflow and outflow to its present value using a discount rate that reflects the risk level of the investment or project, then calculates the total sum of these discounted values. This approach is different from a partial budget model, which does not evaluate NPV.(Barry and Ellinger, 2012) The discount rate used for NPV calculation is based on the cost of capital for an underlying business, and is estimated using the weighted average rates for debt and equity. For agricultural enterprises, the cost of debt can be proxied by farm loan interest rates, which are currently around 8% to 9%.(Federal Reserve Bank of Kansas City, 2024) The cost of equity is more difficult to determine, but tends to be higher than the cost of debt.(Myers, 1984) For this study, a total discount rate of 20% was used based on experimental evidence from farmers given the choice between different payout scenarios.(Duquette et al., 2012).

Compared to relying on nominal dollar values for distant future projections, NPV offers a more precise and analytically robust evaluation, as it accounts for the time value of money and adjusts future cash flows accordingly. Long-term investments with positive net expected returns result in a positive NPV, and when comparing multiple projects, those with higher NPVs are more preferred. We then utilize a mixture of stochastic and deterministic revenue and cost equations input into spreadsheet software to formulate expected NPV solutions. Finally, because some revenue and cost factors are input as stochastic variables (e.g., culling rates and death loss), comparisons between strategies are derived based on the cumulative distributional differences in the NPV (ΔNPV = NPV of BSE less NPV of NBSE) generated from 500 Monte Carlo simulations of 50-yr planning horizons. Details of these major features of the capital budgeting methodology are as follows:

### Revenue Model

The sum of the T period net cashflows from cow/calf operations for BSE and NBSE strategies are $R_{T}=\sum_{c}^{}\sum_{t=0}^{T}\left(q_{ct}p_{ct}-r_{ct}-m_{ct}\right)$ where there are realized sales for each annualized time-period t∈[0,1,2,,,T] at respective prices ($p_{ct}$) and total quantities ($q_{ct}$), within each animal class c∈{calf, cow, bull} less each respective class’s replacement ($r_{ct}$) costs (excluding calves), and annual production-maintenance costs ($m_{ct}$) for bulls. The subsequent NPV for the future cashflow series is therefore $NPV_{0}=\sum_{t=0}^{T}\frac{R_{t}}{{\left(1+i\right)}^{t}}$, where i is the respective discount rate of 20% based on published values for agricultural enterprises.(Duquette et al., 2012) The difference in NPV is $\Delta NPV=NPV_{0}^{BSE}-NPV_{0}^{NBSE}$; for ΔNPV≥0, it must be the case that $R_{T}^{BSE}\geq R_{T}^{NBSE}$. Given veterinarian services and replacement costs of bulls are necessarily larger for the BSE strategy, the only way for this strategy to be preferred by the producer is if the sum of the revenues from calf and cull cow/bull sales is sufficiently larger than its additional veterinarian and replacement costs.

### Number of Cows Conceiving in Each Estrus Cycle

The focus of the model is to assess the effect of differences in bull fertility on herd conception rates, independent of fluctuations in cow fertility. Therefore, if cows have recovered from calving and are actively having estrus cycles, bull fertility is assumed to be the rate limiting factor for the incidence rate of conception per estrus cycle and is therefore considered equivalent with herd fertility in this model. In other words, as long as a cow, or group of cows, are eligible to breed (i.e., have achieved postpartum uterine involution and are having active estrus cycles), the incidence rate of conception per estrus cycle is expressed as bull percentage fertility. As such, the number of cows impregnated per estrus cycle in a given year ($IC_{kt}$) is determined by the size of the cow herd (CH), the number of cows already impregnated in the same year ($\sum_{0}^{k-1}IC_{kt}$), the number of cows within that herd that are eligible to be bred ($e^{cow}$), and by bull percentage fertility ($f^{bull}$). For any predetermined annual breeding season of K estrus cycles in length, the total number of cows impregnated in a given year is determined by the statement, $IC_{Kt}=\left(CH-\sum_{0}^{k-1}IC_{kt}\right)e_{k}^{cow}f^{bull}$. Cow eligibility for breeding is assumed to be 100% in each estrus cycle of the first year, and in each cycle of subsequent years is derived by the statement, $e_{k}^{cow}=\left\{ \begin{array}{lll} & \frac{CH-\sum_{k+2}^{K}IC_{k(t-1)}}{CH}+\;\frac{IC_{k+2(t-1)}}{2CH},\;if\;\frac{CH-\sum_{k+2}^{K}IC_{k(t-1)}}{CH}\; & else\;1\; \end{array}\right.<1$, which is conditional on the number of cows impregnated in the k+2 cycle, and following cycles, of the previous year. This statement imposes a post-partum anestrus period 2 ½ estrus cycles in duration, to the effect that cows bred in a given cycle 1 yr are not eligible to breed the next year until after they pass the postpartum anestrus period. For example, cows bred in the 1^st^ and 2^nd^ estrus cycles of year 1 are eligible to be bred in the 1^st^ cycle of year 2, but only half of all cows bred in the 3^rd^ cycle of year 1 are eligible to breed in the 1^st^ cycle of year 2. The reasoning for the lagged structure is only ~50% of cows bred in cycle k+2 of the previous year are expected to be receptive to breeding during cycle k of the current year, and that no cows bred in any cycle after k+2 of the previous year are expected to be receptive.(Short et al., 1990)

Maximum bull percentage fertility (i.e., incidence rate of conception when $e_{k}^{cow}=1$) is held constant at 66% for the BSE strategy. This value is within the range of what is normally expected for healthy, reproductively sound beef cattle.(BonDurant, 2007) The incidence rate of conception for the NBSE strategy is varied by increments of 1%, and in all scenarios is either equal to, or less than the incidence rate for the BSE strategy; the maximum evaluated difference in incidence rate of conception between strategies is 12% (BonDurant, 2007) This methodology assumes that the presence of subfertile bulls can decrease the incidence rate of conception, but that removing subfertile bulls cannot improve the incidence rate of conception beyond what is normally expected as the biological maximum. The deterministic outcomes for average number of cows bred over the full planning horizon and for the full ranges of breeding season lengths and bull fertilities used in the simulation are reported in results.

### Number of Calves Born and Weaned

For any predetermined annual breeding season of *K* estrus cycles in length, the per period calf revenue is $q_{calft}p_{calft}=\underset{k=1}{\overset{K}{\sum }}\;\left(IC_{kt}\alpha \delta \right){w^{\prime }}_{kt}p_{Wk}$. The number of live calves produced and weaned in each cycle is $IC_{kt}\alpha \delta$, where IC is the number of cows impregnated during the $k^{th}$ cycle, α are the number of remaining fetuses not aborted after pregnancy diagnosis (1−%abortion), and δ are the number of calves that did not die after birth (1−%deathloss). Because the objective of this study is to evaluate the effects of the BSE on reproductive efficiency and fertility, the abortion rate and mortality rate for calves were assumed to be the same for both strategies.

### Average Weaning Weights of Calves

The average weaning weight of the calves conceived in each cycle is a row vector ${w^{\prime }}_{kt}=b+ADG\left(age_{kt}\right)$, where b and ADG are an assumed average birth weight and average daily gain (see Table 1), while the average age of weaned calves conceived within the $k^{th}$ cycle is $age_{kt}=\left\{ \begin{array}{l} 225\;-\;10.5,\;\;if\;k=1\;age_{\left(k-1\right)t}\;-\;21,\;if\;k\geq 2\; \end{array}\right.$. This equation assumes that the average age of calves born in any given estrous cycle is the age of a calf born in the middle of that estrus cycle (i.e., 10.5 d after the beginning of the estrous cycle), and that the average length of a bovine estrus cycle is 21 d.(Senger, 2003) Therefore, the total weight of calves sold for the year is $\sum_{k=1}^{K}\left(IC_{kt}\alpha \delta \right){w^{\prime }}_{kt}$. Finally, the total calf revenue for each production period is derived by multiplying the total weight sold per cycle by a corresponding column vector of future prices per unit of weight, $p_{Wk}$, for the respective weight classes that the average weight of the calves falls in per cycle. For example, if $W=[\begin{array}{llll} 250-300,\; & 301-350 & ... & 701-751\; \end{array}]$, then $p_{Wk}=\left[\begin{array}{l} 2.40\;2.41\;...\;1.84\; \end{array}\right]$. It is well known that price per unit of weight is generally decreasing in average live weight, commonly called a price slide. Table 2 shows the price slide used to calculate calf revenue. Note, the price/weight class relationship for the future cash flows is held constant over production periods for this analysis. The deterministic outcomes for average weaning weights and calf revenues over the full planning horizon and for the full ranges of breeding season lengths and bull fertilities used in the simulation are reported in results.

**Table 2.** Price slide used to calculate revenue from calves weaned

Calf Weight (Lb)	$/Lb^1^	Calf Weight (kg)	$/kg
250	2.40	114	5.28
300	2.41	136	5.30
350	2.37	159	5.21
400	2.30	182	5.06
450	2.23	205	4.91
500	2.15	227	4.73
550	2.09	250	4.60
600	2.01	273	4.42
650	1.94	295	4.27
700	1.84	318	4.05

^1^Aggregate weekly average from MS steers and heifers 2023, Source: USDA/AMS.

### Number of Cows and Bulls Culled

For the expected culling and mortality outcomes for any given production year, we estimate the total number of animals culled based on the probability of an individual cow or bull being culled for specific reasons (e.g., aging out of the program, udder confirmation, injury/attitude, failing a BSE, or dying). The exception to this rule is for the number of cows that are culled for being diagnosed as non-pregnant at the end of the breeding season, which is based on the deterministic formulas described above. To ensure that no cow or bull can be simultaneously selected for more than one culling event, culling events are made sequentially. The total number of cows culled is determined through a combination of deterministic and stochastic factors, and the methodology for determining the number of cows being randomly culled within each production period will be presented after outlining the determination of revenue from culling cows. The realized per period total revenue for culled cows is $q_{cow\;t}p_{cow\;t}=CC_{t}w_{cow}p_{cow}$, where the total number of cows culled is $CC_{t}=\underset{}{\underset{not\;impregnated}{\underbrace{\left(CH-\underset{k=i}{\overset{K}{\sum }}\;IC_{t}\right)}}\;}+\underset{}{\underset{random\;nonreproductive\;reasons}{\underbrace{NR_{cowt}}}}\;\;$, where $w_{cow}$ is an assumed average weight of a mature cow for a particular herd, and $p_{cow}$ is the projected salvage price per unit of weight for slaughter cows, both held constant for NPV analyses (see Table 1). The number of cows culled out of the herd for nonreproductive reasons is treated as an independent random event per period. We assume the average working life span of a cow is 10 yr for either strategy, and that the probability of a cow being culled for non-reproductive reasons is the inverse of the average working life span. However, only cows that are pregnant are eligible to be culled for nonreproductive reasons. Because the number of impregnated cows may vary, the pool of cows eligible to be culled for nonreproductive reasons may also vary.

**Figure 1.**
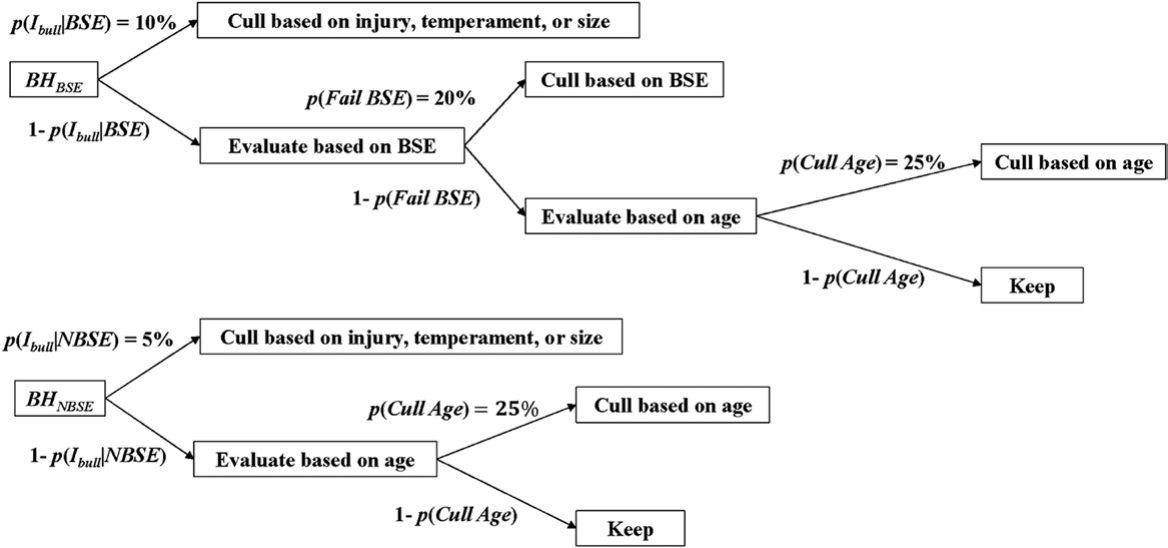
Order of operations for bull culling events for BSE strategy and NBSE strategy. Each boxed variable represents a quantity of bulls within a cohort, and each arrow represents the discreet, binomial probability of a bull being partitioned to each cohort.

Given the reasons bulls may be culled are entirely random, the methodology for determining the number of bulls culled within each production period will be discussed after addressing the derivation of cull bull revenue. The realized per period total revenue for culled bulls is $q_{bullt}p_{bullt}=CB_{t}w_{bull}p_{bull}$. The total number of bulls culled during any given period conditional on conducting BSE testing or not is $CB_{t}=\left\{ \begin{array}{lll} & \underset{random\;injury}{\underbrace{I_{bull\;t}}}+\underset{random\;fail}{\underbrace{Test_{bull\;t}}}+\underset{random\;age\;reasons}{\underbrace{Age_{bullt}}}if\;BSE\; & \underset{random\;injury}{\underbrace{I_{bull\;t}}}+\underset{random\;age\;reasons}{\underbrace{Age_{bull\;t}}}if\;NBSE\; \end{array}\right.$. Like for cull cow revenues, the average weight and salvage price per unit of weight for slaughter bulls is held constant for NPV analyses. The total number of bulls being culled in any given production period is conditional on whether a BSE test is conducted or not. For bull culling events, the discrete quantity of bulls culled for each event in any period is dependent on the number of bulls eligible to be culled for each event and the base-line probability for each culling event. The initial number of bulls eligible to be culled each year is the total bull herd (BH). The number of bulls eligible to be culled for subsequent culling events is determined by subtracting the number of bulls culled in the previous culling event from the number of bulls that were eligible in the previous event. Figure 1 provides the decision tree and associated probabilities related to bull culling events for each strategy.

As can be seen in Figure 1, culling events are based on injury and other random factors (e.g., temperament, size, etc.) first for both strategies, and only bulls that are not culled in the first culling event are eligible to be culled based on subsequent criteria. It is assumed that the expected number of culls due to injury, temperament, or size $E[I_{bull\;\;\;t}|BSE]>E\left[I_{bull\;\;\;t}|NBSE\right]$, as testing bulls requires more handling and thus increases the likelihood of injury and/or manifestation of aggressive behavior. It is assumed that approximately 20% of bulls presented for BSE will be categorized as unsatisfactory and subsequently culled.(Barth and Waldner, 2002; Barth, 2018) Because BSE testing will necessarily increase the likelihood of culling more bulls per period due to infertility than not testing, the $E[CB_{t}|BSE]>E[CB_{t}|NBSE]$. It follows that the $E\left[q_{bull\;t}p_{bull\;\;t}|BSE\right]>E\left[q_{bull\;t}p_{bull\;t}|NBSE\right]$, resulting in increases in total per period cull revenues by conducting BSE testing, but also increases in bull replacement costs. The stochastic outcomes for number of bulls culled over 500 iterations of the Monte Carlo simulation are presented in results. The culling rates for both strategies are independent of assumed differences in fertility between strategies.

For the BSE strategy, only bulls that pass the BSE can be culled based on age. For the NBSE strategy, the only subsequent culling decision after injury, temperament, and size is culling based on age. Culling events based on age assumed the average working life span of a bull is 4 yr for either strategy; the probability of a bull being culled based on age is the inverse of the average working life span.

To complete the derivation of per period cow and bull revenues, the general methodology for deriving the random number of culled cows and bulls is now addressed. For the current NPV analysis, the number of culled (C_) cows (CC) or culled bulls (CB) for each culling event each period $ce\in \left\{NR_{cow},Test_{bull},I_{bull},Age_{bull}\right\}$ are independently determined from a Binomial distribution describing the probability a particular number of cows or bulls will be culled (a.k.a. successes) out of a required herd size maintained (_H) (a.k.a. trials) for cows (CH) or bulls (BH), given a time invariant contingent culling event probability (p(ce)) that any given individual is independently and identically selected to be culled out of _H. All probabilistic culling events are independently and identically drawn across all T periods. A general depiction of the various Binomial probability mass functions is $f\left(C\_,\_H,p\left(ce\right)\right)=\mathrm{Pr}\left(X=C\_\right)=\left(\begin{array}{l} \_H\;C\_\; \end{array}\right){\left(\;p\left(ce\right)\right)}^{C\_}{\left(1-p\left(ce\right)\right)}^{\_H-C\_},$ with mean E[X]=_Hp(ce) and variance Var(X)=_Hp(ce)(1−p(ce)). For each production period, the number of animals C_ culled per event is independently and identically drawn from f(C,H,p(ce)), and for bulls is also conditional on whether the producer utilizes BSE or not.

The distribution of the total number of bulls culled in any given period $CB_{t}$ is the sum of the independent Binomial distributions. Because p(ce) are not necessarily equal, the total number of bulls culled in sequence is best described as a Poisson binomial distribution with mean $\mu =BHp\left(I_{bull}\right)+BH^{\prime }p\left(Test_{bull}\right)+BH^{\prime \prime }p\left(Age_{bull}\right)$ and variance $\sigma^{2}=BHp\left(I_{bull}\right)\left(1-p\left(I_{bull}\right)\right)+BH^{\prime }p\left(Test_{bull}\right)$$\left(1-p\left(Test_{bull}\right)\right)+BH^{\prime \prime }p\left(Age_{bull}\right)\left(1-p\left(Age_{bull}\right)\right)$, where BH^′^ and BH^″^ are bulls that remain in the bull herd after each probabilistic event. However, the derivation of the distribution for the total number of culled cows is not as straightforward as the distribution arising from those not impregnated within any given production period $\underset{not\;\;impregnated}{\underbrace{\left(CH-\underset{k=i}{\overset{K}{\sum }}\;IC_{t}\right)}}$ is not well understood and may be production period and number of cycles dependent on multiple probabilistic events, but can be observed via simulation.

### Maintenance and Replacement Costs

Production-maintenance and replacement costs in the total net returns equation are now detailed. We estimate maintenance costs (i.e., feed, yardage, vaccines, etc.) based on the number of bulls needed for each strategy, and replacement costs based on the number of replacement cows and bulls needed to purchase each year. Because some scenarios use different cow:bull ratios, the maintenance costs for the entire bull battery may vary between strategies. However, we assume maintenance costs for the total cow herd are the same for both strategies. We acknowledge that there may be variation in the costs associated with producing calves with varying weight distributions. However, we assume that output prices on a sliding scale represent breakeven points relative to costs incurred to produce various weights of calves, and for this reason we make no attempt to simulate differences in costs associated with varying calf weight distributions.

Bull maintenance costs are $m_{ct}=BBc_{bull}$, where the future per period costs $c_{bull}$ are treated as time invariant for derivation of the NPV. However, there is an annual per bull veterinary cost associated with performing the BSE of $100, such that $E\left[c_{bull}|BSE\right]>E\left[c_{bull}|NBSE\right]$. In contrast, replacement costs are expected to vary over the planning horizon due to the random nature of the number of cows and bulls being culled each production period. As such, the expected total per period replacement costs are $r_{cowt}+r_{bullt}=\left(E\left[CC_{t}\right]+E\left[CD\right]\right)r_{cow}+\left(E\left[CB_{t}\right]+E\left[BD\right]\right)r_{bull},$ where future per head prices $r_{cow}$ and $r_{bull}$ are treated as time invariant. Note, because more bulls are being culled by testing, the expected replacement costs are also greater, $E\left[r_{bull\;\;\;t}|BSE\right]>E\left[r_{bull\;\;\;t}|NBSE\right]$. Therefore, the net effect of performing BSE on period total revenues is dependent on the expectations of future cull rates and replacement cost assumptions for new bulls.

All bulls and cows in the herd are eligible for mortality events. Mortality events are used to determine the number of bulls and cows needed to purchase but are not factored into culling decisions. For the scenarios in this study, the probability of bull and cow mortality events is assumed to be the same for both strategies. The number of bull and cow mortality events are also drawn from a binomial distribution. The model assumes that bulls and cows are immediately replaced following mortality events so that each period starts with the same number of animals, thus accounting for the cost of replacements.

### Monte Carlo Simulation Methodology

From historical observation, stochastic events are expected to occur throughout the future financial planning horizon that impact either costs and/or revenues. The culmination (convolution) of multiple random events generates the NPV and its probability density functions. The complexity of deriving the NPV density function increases as the number of random events increase, even if all random event distributions are known with certainty.(Jäckel, 2002) For a variety of financial decisions, including capital budgeting and NPV comparisons, Monte Carlo simulation has a long history and is a common methodology for making multi-period financial decisions to account for numerous known and expected future stochastic events.(Salazar and Sen, 1968; Dhiensiri and Balsara, 2002; Jäckel, 2002; Mun, 2006; Clark et al., 2010) From the Monte Carlo simulation of future capital budgets in this study, an estimate of the NPV probability density functions for each decision strategy are generated. From the generated probability density functions, strategy means and variances provide information as to which strategy is expected to be more/less profitable and more/less risky.

The generated NPV probability density function is dependent on future probability distribution functions of multiple individual events in the future, which are unlikely known with certainty at the time of decision making. Therefore, each event probability distribution function must either be estimated from prior information or assumed in the absence of priors. A well-known concern of Monte Carlo analyses is that the reliability of precise estimates of the future is dependent upon the difference between the estimated and realized individual random event distributions in the future. However, this analysis reports qualitative differences between strategies where these results are expected to be minimally impacted by the accuracy of the assumed individual random event distributions described earlier.

Monte Carlo simulations provide NPV numerical experiments for each strategy {BSE and NBSE} controlling for plausible incremental fertility differences [1%,…,12%], as well as management decisions regarding breeding season length [2,3,4,5,6]. To control for management decisions regarding the pressure placed on each bull, two overarching numerical experiments are conducted for the aforementioned strategies and controls, first assuming a cow:bull ratio of 25:1 and again assuming a ratio of 33:1.

Increasing the number of iterations of random draws from multiple individual distributions increases the stability and convergence of the targeted outcome distribution.(Jäckel, 2002) For this analysis, 500 iterations of NPVs were found to provide ample stability for all NPV density functions. Next, the preference for performing BSE or not is contingent on various controls derived from the $\Delta NPV_{i}=NPV_{BSE,i}-NPV_{NBSE,i}$ from i=[1,...,500] iterations of a 50-period planning horizon. The ultimate financial comparison between BSE and NBSE strategies are based on the differences between the estimated cumulative probability distribution functions of the $\Delta NPV_{i}\geq 0$ indicating the likelihood, under various controls, of whether the BSE strategy is financially preferred to the NBSE strategy.

### Rationale for Production Scenarios Evaluated

After establishing the baseline capital budget, various production scenarios related to differences in breeding season length, and cow:bull ratios can be evaluated. Calf output as a result of breeding season length is input in units of numbers of estrous cycles in whole number values. We evaluate breeding season lengths as short as 2 estrus cycles, because this is typically the shortest breeding season most producers will attempt. We evaluate breeding seasons as long as 6 estrus cycles because at that point the breeding season begins to approximate leaving the bulls in the cow herd year around, and the differences observed between each additional estrus cycle unit of length become progressively smaller. We evaluate cow:bull ratios of 25:1 and 33:1 because they are comparable to cow:bull ratios in other studies.(Wiltbank and Parish, 1986; Menegassi et al., 2011) In the study by Menegassi et al. (2011) one of the chief financial benefits of implementing the BSE was that it enabled herds to run a higher cow:bull ratio; the next highest cow:bull ratio typically employed after 25:1 was 33:1 (i.e., 4 bulls per 100 cows vs. 3 bulls for 100 cows).

For each breeding season length, results from the Monte Carlo simulation allow for comparisons between the two strategies based on the distribution of proportions of iterations where ΔNPV > 0 (BSE strategy preferred to NBSE strategy) depending on the level of possible fertility differences. These proportions are plotted as density curves of cumulative probabilities over the full range of absolute differences in fertility from not using the BSE, and are simulated for scenarios where the cow:bull ratio is 25:1 for both strategies, and where it is 33:1 for the BSE strategy and 25:1 for the NBSE strategy. The point of indifference between the two strategies occurs at the difference in fertility where the cumulative probability of ΔNPV > 0 is 50%.

## RESULTS

**Figure 2.**
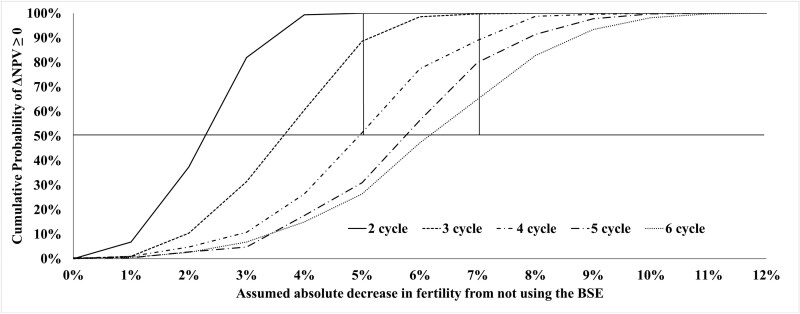
The cumulative probability curves for the likelihood that the BSE strategy has a higher NPV than the NBSE strategy. Each curve represents a different breeding season length in units of estrus cycles, and assumes that both strategies use a cow:bull ratio of 25:1. The vertical lines represent differences in fertility of 5% & 7%, which is approximately the range of expected differences in incidence rate of conception per cycle between herds that use BSE and those that don’t based on published literature.(Wiltbank and Parish, 1986 DOI: 10.1016/0093-691X(86)90093-2) The horizontal line represents the point at which it is equally likely that using the BSE will have a higher NPV than not using the BSE.

**Figure 3.**
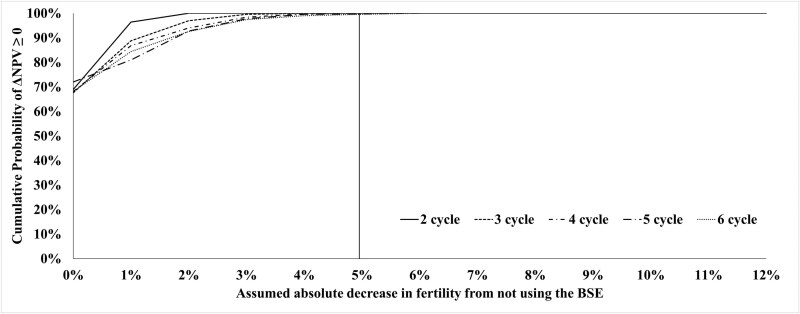
The cumulative probability curves for the likelihood that the BSE strategy has a higher NPV than the NBSE strategy. Each curve represents a different breeding season length in units of estrus cycles and assumes that the BSE strategy uses a cow:bull ratio of 33:1 versus a ratio of 25:1 for the NBSE strategy. The vertical line represents an absolute decrease in fertility of 5% from not using the BSE strategy and is the difference at which the cumulative probability of the BSE strategy having a higher NPV is essentially 100%.

Figure 2 displays the cumulative probability density curves for the scenario where the cow:bull ratio for both strategies is 25:1 across a range of assumed absolute decreases in fertility from not using the BSE; each curve represents a different breeding season length. These results indicate that if both strategies have the same cow:bull ratio, for any given difference in fertility from not using the BSE, the probability the BSE strategy is more financially attractive (i.e., ΔNPV > 0) decreases with increasing breeding season length. Figure 3 displays the same curves when the cow:bull ratio is 33:1 for the BSE strategy and 25:1 for the NBSE strategy. These results indicate that the BSE is more likely to be strictly more profitable at higher cow:bull ratios.

An explanation for the results observed in Figures 2 and 3 are as follows. The average numbers of cows bred, weaning weights, and revenues from calves over the 50-yr planning horizon for each breeding season length of K estrus cycles and each $f^{bull}$ from 54% to 66% are reported in Table 3. These values explain a large portion of the results observed in Figures 2 and 3. Decreasing bull fertility results in fewer calves born, and more calves born later in the year, thereby decreasing weaning weights and revenues from calf sales. The effect of bull fertility on average weaning weights becomes more pronounced as breeding season length increases. While increasing breeding season length increases the opportunities for cows to be impregnated and can thereby offset some of the effect of poor bull fertility by producing more pregnancies than would have resulted from a shorter breeding season, increasing breeding season length also decreases average weaning weight. Though the effect of decreasing weaning weights is made up for to some degree by the price slide, the heavier calves will always be more valuable, providing a rationale for maintaining shorter breeding seasons.

**Table 3.** Average numbers of cows bred, weaning weights, and calf revenues over the 50-yr planning horizon for each breeding season K estrus cycles in length and each bull fertility value $f^{bull}$ from 54% to 66%

*K*	*f* ^bull^												
	66%	65%	64%	63%	62%	61%	60%	59%	58%	57%	56%	55%	54%
Average numbers of cows bred													
2	885	878	871	864	856	848	840	832	824	816	807	798	789
3	958	955	950	946	942	937	932	927	921	915	909	903	897
4	983	981	979	976	973	970	967	964	960	956	952	948	943
5	993	992	990	989	987	985	983	980	978	976	972	969	966
6	998	997	996	995	995	993	992	991	990	988	986	984	982
Average weaning weights of calves (Lb/kg)													
2	498/226	498/226	498/226	498/226	497/226	497/226	497/226	497/226	497/226	496/225	496/225	496/225	496/225
3	491/223	491/223	490/223	490/223	489/222	489/222	488/222	488/222	487/221	487/221	486/221	486/221	485/220
4	486/221	485/220	484/220	483/220	482/219	481/218	480/218	479/218	479/218	478/217	477/217	476/216	475/216
5	482/219	480/218	479/218	478/217	477/217	475/216	474/215	473/215	471/214	470/214	469/213	468/213	466/212
6	479/218	478/217	476/216	475/216	473/215	471/214	470/4214	468/213	466/212	464/211	463/210	461/210	459/209
Average revenues from calves (Thousands of $)													
2	937	930	922	914	906	897	888	880	871	862	853	843	833
3	1,006	1,002	996	991	986	980	974	969	962	955	948	941	934
4	1,024	1,021	1,017	1,013	1,009	1,005	1,001	996	991	986	980	975	969
5	1,028	1,,025	1,022	1,019	1,015	1,011	1,007	1,002	998	994	989	984	979
6	1,030	1,027	1,023	1,020	1,017	1,013	1,009	1,005	1,001	997	992	988	982

^1^Microsoft Excel, Microsoft Corporation, Redmond, WA

The averages and standard deviation for number of bulls culled per year over 500 iterations of the Monte Carlo simulation were 12.6 (0.5) for the BSE strategy and 9.5 (0.4) for the NBSE strategy. In effect, this means that a strategy that employs the use of BSE will necessarily result in the operation having to purchase an additional 2 to 3 bulls per year relative to the NBSE strategy. In that regard, the replacement cost of $7,500 per bull and associated depreciation is by far the largest expense associated with performing BSE; it is a full order of magnitude higher than the cost associated with paying a veterinarian to perform the BSE.

## DISCUSSION

Whereas the results show that the use of BSE provides financial benefits to producers overall, our findings also provide plausible financial explanations for why not all producers choose to utilize the BSE despite its demonstrated benefits. For example, the Mississippi survey found that cow-calf producers who leave their bulls out with the cows all year are less likely to hire a veterinarian to perform BSE on their bulls. The current study suggests that the likelihood of receiving sufficient financial rewards from the BSE diminishes with increasing breeding season length, which could explain the results seen in the survey.

Regarding how much of an increase in reproductive efficiency a producer might reasonably expect from performing BSE, it is difficult to make an exact estimate. An oft cited study by Wiltbank and Parish (1986) found an overall improvement in pregnancy rates of ~5% to 6% when Santa Gertrudis cows and heifers were bred to bulls that had been screened for sub-fertility using semen criteria similar to the current SFT standards. This improvement in pregnancy rate was in comparison to cohorts of animals from the same population that were bred in the same year to bulls that were representative of the population of bulls prior to screening; all breeding groups had a cow:bull ratio of ~25:1.(Wiltbank and Parish, 1986) The effect of screening bull batteries on incidence rate of conception per cycle in the aforementioned study varied slightly, as in the first year the groups were exposed to the bulls for 120 d, and in the second year they were exposed for 90 d. In both cases, the improvement in pregnancy rate was ~5% to 6% (i.e., Increase from 87% to 93% in year 1, and 85% to 90/91% in year 2). On average, these improvements represented a difference in incidence rate of conception per cycle of ~8% for year 1, and ~7% for year 2. It should be noted that the baseline incidence rate of conception per cycle for the herds in this study was quite low to begin with (<40%), and the benefits were determined by implementing the BSE and thereby improving conception rates. In herds where baseline incidence rate of conception is closer to the industry standard (i.e., ~60%), it may be harder to realize similar gains in incidence rate of conception by implementing the BSE. Nevertheless, the vertical lines in Figures 2 and 3 highlight the regions where we might expect to see differences in fertility due to not using the BSE under field conditions.

In this study, we did not vary the baseline setting for useful life of the bulls between strategies. However, the algorithms for culling were not equivalent for both strategies, and while the independent probability of being culled based solely on age was the same, the number of bulls available to be culled due to age was not. This resulted in fewer bulls being culled due to age in BSE strategy, but more bulls being culled overall. Menegassi et al. (2011) observed that herds that did not use the BSE tended to keep bulls in the herd longer and had an average useful life span of 6.2 yr instead of 4 yr for herds that did use the BSE. These observed life spans also resulted in a higher overall cull rate for herds that used the BSE, which is consistent with the current study.

The planning horizon and discount rate used in this study were on the upper end of what might be expected for an agricultural operation. In reality, the effect of implementing BSEs in a herd would likely be realized within a few years, and a much shorter planning horizon may be sufficient. However, while a sensitivity analysis revealed that a shorter planning horizon (i.e., 15 yr) and lower discount rate (i.e., 5%) did not substantially change the results of the model, we did not know if this would be the case a priori, and the results of the model were reported based on the original parameters.

The model assumed that roughly 1 in 20 bulls would be culled due to injury, temperament, or size in any given year if BSE was not performed, and that if the BSE was performed the odds would be approximately 1 in 10. In reality, performing the BSE may not significantly increase the odds of culling due to the factors, and the authors are unaware of any published data that demonstrate a difference in non-BSE related cull risk due to the implementation of the BSE. However, the possibility that there might be a difference in risk was included to steel man the model against the argument that producers might not choose to implement the BSE due to the added risk of gathering and processing bulls for the examination. Bringing bulls into a handling facility for BSE is a stressful event which may precipitate aggressive behavior and/or injury, and may also indicate to a producer that a bull is oversized if it is has difficulty moving through alleyways and chutes. However, if the risk of non-BSE related culling between the two strategies were equivalent, then any change in the results of the model would favor the BSE strategy being more profitable. Notwithstanding, a sensitivity analysis where the difference in risk of injury was eliminated did not yield substantially different results.

The findings from this study are based on the assumption that the primary incentive for management decisions is profit. However, there may be other factors aside from profit that dissuade producers from using the BSE, and these factors in turn may also affect variables such as cow fertility in ways that we could not assess in this model. For example, in the survey of Mississippi cow-calf producers, the most stated reason for not hiring a veterinarian to perform BSE was not having enough time/help to do so. The survey also found that 8% of respondents owned cattle as a primary source of income, 41% of respondents indicated that cattle sales were not a primary, but still significant source of income, and that 82% indicated a reason they owned cattle was because they enjoyed caring for cattle and the lifestyle associated with it.(Gunderson et al., 2024) While owning cattle for profit and for enjoyment are not mutually exclusive states of the world, the survey results do suggest that there are a substantial number of producers who own cattle more for enjoyment than for profit. As such, a producer that falls into this latter category may not be as willing to incur the expense and hassle of hiring a veterinarian to perform BSE based solely on improving profitability.

Another important possible producer benefit not controlled for in this study was that of having a close working relationship with a veterinarian, who not only performs BSE, but also provides consultation regarding control of morbidity, mortality, and pregnancy retention. The capital budgeting model allows for these variables to be assessed, but their effect on the profitability of performing BSE was not evaluated as part of this study and were assumed to be equivalent between strategies. Lastly, the current study also assumes that the only meaningful differences in conception rates would be due to using the BSE, or not using the BSE, whereas the implementation of a strategy to hire a veterinarian to perform BSE, or the lack thereof, may be correlated to other management strategies that could also affect conception rates (e.g., via improvements in cow fertility).

## CONCLUSIONS

As breeding season length decreases, the probability of the BSE being financially viable increases. However, if the BSE enables a cow-calf producer to run a higher cow:bull ratio (e.g., 33:1 with the BSE versus 25:1 without), then the BSE is more likely than not to be profitable, even if the producer realizes no increase in fertility from the BSE.
